# A comprehensive evaluation of the impact of telemonitoring in patients with long-term conditions and social care needs: protocol for the whole systems demonstrator cluster randomised trial

**DOI:** 10.1186/1472-6963-11-184

**Published:** 2011-08-05

**Authors:** Peter Bower, Martin Cartwright, Shashivadan P Hirani, James Barlow, Jane Hendy, Martin Knapp, Catherine Henderson, Anne Rogers, Caroline Sanders, Martin Bardsley, Adam Steventon, Raymond Fitzpatrick, Helen Doll, Stanton Newman

**Affiliations:** 1 Health Sciences Research Group, University of Manchester, Manchester, UK; 2 School of Community and Health Sciences, City University, London, UK; 3 Imperial College Business School, Imperial College London, London, UK; 4 Personal Social Services Research Unit (PSSRU), London School of Economics and Political Science, London, UK; 5 Institute of Psychiatry, King's College London, London, UK; 6 Nuffield Trust, London, UK; 7 Department of Public Health, University of Oxford, Oxford, UK

## Abstract

**Background:**

It is expected that increased demands on services will result from expanding numbers of older people with long-term conditions and social care needs. There is significant interest in the potential for technology to reduce utilisation of health services in these patient populations, including telecare (the remote, automatic and passive monitoring of changes in an individual's condition or lifestyle) and telehealth (the remote exchange of data between a patient and health care professional). The potential of telehealth and telecare technology to improve care and reduce costs is limited by a lack of rigorous evidence of actual impact.

**Methods/Design:**

We are conducting a large scale, multi-site study of the implementation, impact and acceptability of these new technologies. A major part of the evaluation is a cluster-randomised controlled trial of telehealth and telecare versus usual care in patients with long-term conditions or social care needs. The trial involves a number of outcomes, including health care utilisation and quality of life. We describe the broad evaluation and the methods of the cluster randomised trial

**Discussion:**

If telehealth and telecare technology proves effective, it will provide additional options for health services worldwide to deliver care for populations with high levels of need.

**Trial Registration:**

Current Controlled Trials ISRCTN43002091

## Background

It is expected that increased demands on health and social care services will result from the rise in the numbers of older people with long-term conditions and social care needs [1]. While there are alternative proposals about the implications of increasing numbers of older people on demand for services [2], much planning is predicated on expected increases in social and health service use amongst older people. Shifting the balance of care towards the home environment is seen as requiring an investment in 'upstream' interventions by providing enhanced primary and community care-based alternatives to secondary care and focusing on self-care at the patient level.

### The role of technology

In the current context of economic pressures and a desire to secure efficiency savings, there is significant interest in the potential for technology to reduce utilisation of health services in older people with long-term conditions and social care needs, while improving the quality and cost-effectiveness of care. There are a number of relevant types of telemonitoring technology and a lack of consensus concerning terminology. For the present paper we make the following distinction:

• Telecare is the remote, automatic and passive monitoring of changes in an individual's condition or lifestyle (including emergencies) in order to manage the risks of independent living. Examples include movement sensors, falls sensors, and bed/chair occupancy sensors. These technologies are generally provided to patients with social care needs.

• Telehealth is the remote exchange of data between a patient and health care professional to assist in the diagnosis and management of a health care condition. Examples include blood pressure and blood glucose monitoring. These technologies are generally provided to patients with long-term health conditions such as diabetes.

**Figure 1:**
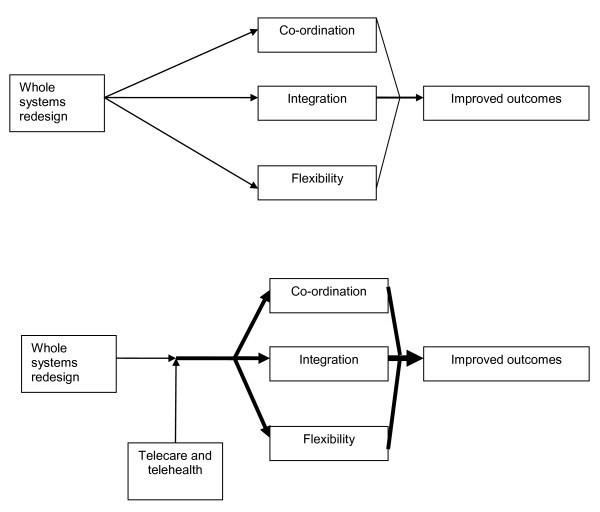
**Telecare and telehealth as an 'effect modifier' of wider system redesign**.

In the United Kingdom, the announcement of the Whole System Demonstrator pilots led to the subsequent award of funding to teams in three areas of England (Kent, Newham and Cornwall) to implement service redesign to support individuals with long-term and complex health and social care needs. This 'whole systems redesign' was designed to create multidisciplinary teams in health and social services and the development of integrated care plans to deliver care more effectively to these patient populations. An important part of the 'whole systems redesign' was the introduction of telemonitoring technology in the home to support the provision of these new services and serve as an 'effect multiplier' for changes in service delivery (See Figure 1). The aim of the Whole System Demonstrator trial is to evaluate whether telehealth for people with long-term conditions and telecare for people with social care needs can provide cost-effective care to improve outcomes, maintain independence, achieve significant gains in quality of life and reduce unnecessary acute hospital use and costs.

### Evidence for the effectiveness and cost-effectiveness of telecare and telehealth

Several reviews of the effectiveness of telecare and telehealth have been published both within specific disease areas [3-5] and across different areas [6,7]. Much of the available literature refers to pilot projects and the assessment of the impact of these devices on short-term outcomes and the majority of studies do not meet robust evaluation standards. Very few of the studies reviewed have assessed the longer-term or routine use of such technologies. A systematic review of 24 trials of interactive health communication applications [8] most closely relate to what is defined here as telehealth. In the review, telehealth had a significant positive effect on knowledge, social support, behavioural outcomes (e.g. calorific intake, exercise and medication taking) and clinical outcomes (e.g. asthma symptoms, HbA1c levels and body mass index). It was not possible to determine whether they had an effect on emotional outcomes or overall healthcare resource use. Another recent systematic review of telecare and telehealth interventions reported an emerging evidence base for the clinical effectiveness of telehealth technologies aimed at vital signs monitoring but insufficient high quality evidence for the effectiveness of telecare applications such as safety and security monitoring [9].

The clear potential of telehealth and telecare technology to improve care and reduce costs combined with the lack of rigorous evidence of actual impact means that a randomised controlled trial is required. The remainder of this paper will describe the protocol for the evaluation of the Whole System Demonstrator pilots and the methodological issues raised in the evaluation.

## Methods/Design

The broad research aim is to assess the effectiveness and cost-effectiveness of (a) telehealth in the management of patients with long-term health conditions, and (b) telecare in the management of patients with social care needs. The proposed trial is a large scale, pragmatic health technology assessment trial, designed to randomise suitably large numbers of patients and assess the impact of a broad class of telemonitoring technologies in the context of routine delivery of NHS care [10,11].

As with any pragmatic trial, the proposed design was a compromise between methodological, ethical and policy issues. The optimal assessment of the cost-effectiveness of a new health technology is a randomised controlled trial, and this was the initial basis for all design discussions. However, subsequent discussions with sites highlighted the importance of designing a trial that had the support of stakeholders, and that individual randomisation of patients was unlikely to be acceptable.

**Figure 2:**
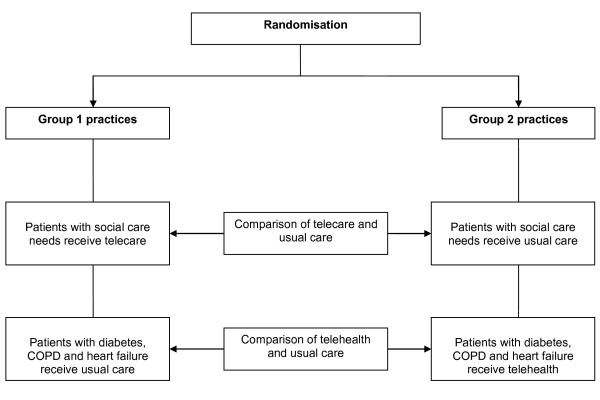
**The basic trial design**.

To deal with these issues, the proposed trial used a cluster randomised trial design (see Figure 2). Although many services provided to patients (especially those with social care needs) are delivered outside primary care, general practices were used as the unit of allocation because they are stable organisations involved in the care of all patients in each site.

General practices were randomised so that eligible patients within their populations would receive access to *one *technology (i.e. *either *telehealth or telecare). Each practice would thus provide intervention participants for one technology (e.g. telehealth) and control participants for the other technology (e.g. telecare) or vice versa. This ensured that equity of access existed at the level of the practice population, and that no practice was asked to risk randomisation to a no-treatment control where all patients would be denied access.^1^

**Figure 3:**
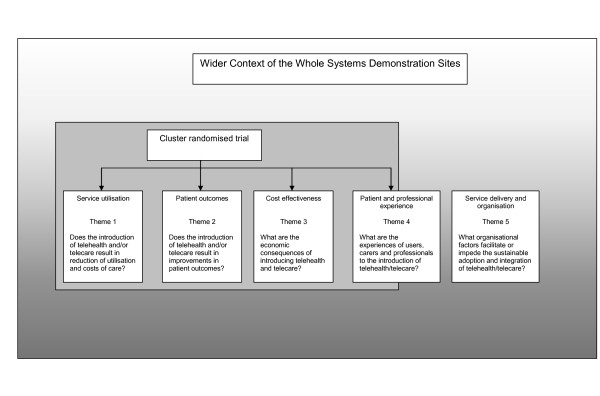
**Research Themes**.

Clearly, the introduction of a complex suite of telemonitoring technologies and the associated service changes raised more questions than could be answered with a conventional trial alone, so that the proposed cluster trial was used as a structure, around which a wider evaluative process was designed in line with current convention about the assessment of complex interventions [12,13]. The broad evaluation structure is outlined in Figure 3, and the research questions listed below.

• *Theme 1: Service utilisation*. Does the introduction of telehealth or telecare result in reduction of service utilisation and costs of care?

• *Theme 2: Clinical effectiveness*. Does the introduction of telehealth or telecare result in improvements in quality of life, well being, self care, and carer burden?

• *Theme 3: Cost-effectiveness*. What are the economic consequences of introducing telehealth and telecare?

• *Theme 4: Patient and professional experience*. What is the experience of service users, carers and health and social care professionals during the introduction of telehealth and telecare?

• *Theme 5: Service delivery and organisation*. What organisational factors facilitate or impede the sustainable adoption and integration of telehealth and telecare?

**Figure 4:**
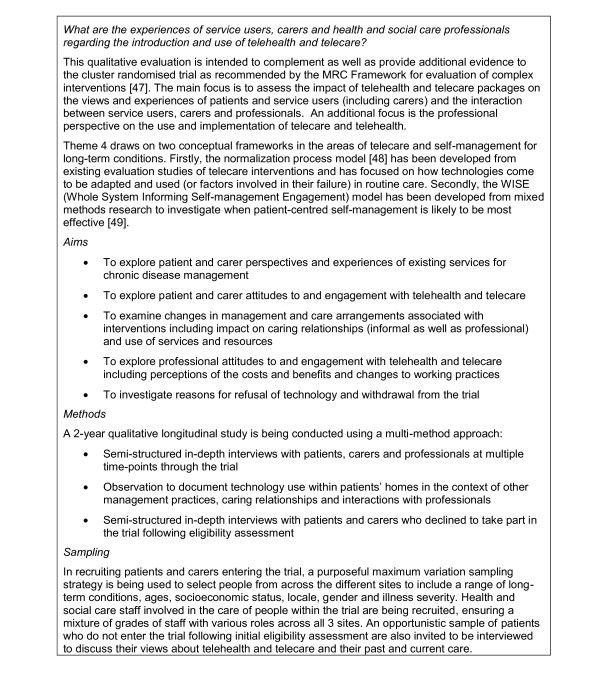
**Theme 4**.

**Figure 5:**
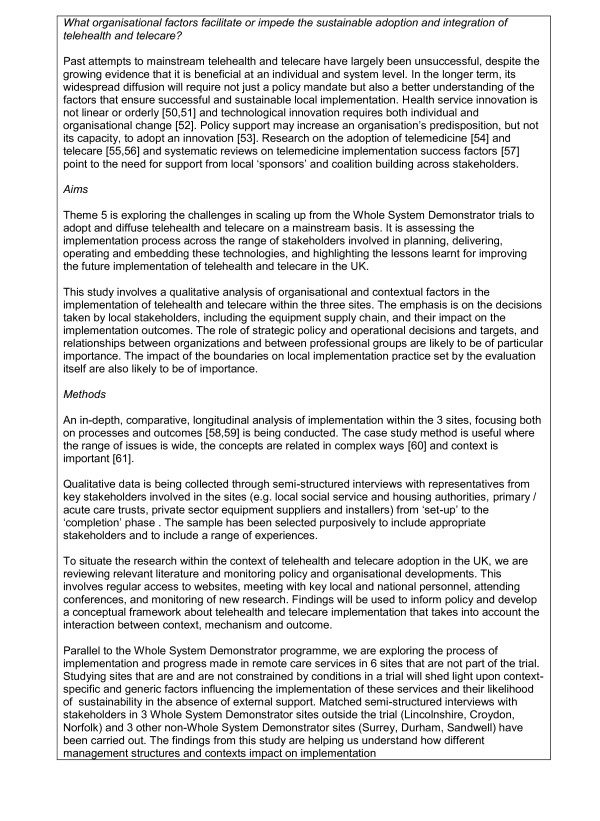
**Theme 5**.

The rest of this report will detail the protocol for the quantitative analysis of the core trial (Themes 1-3). A summary of the related qualitative work (Themes 4 and 5) can be found in Figure 4 and 5.

**Figure 6:**
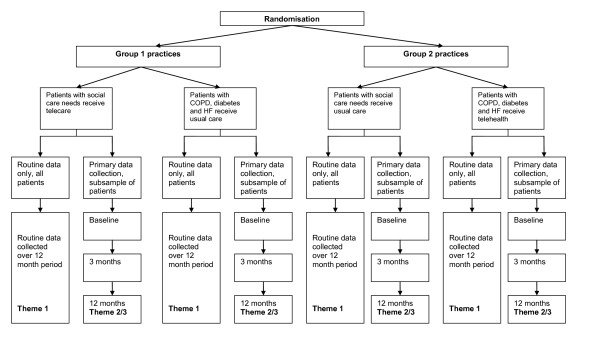
**Detailed study design**.

Themes 1-3 were all based on the same core trial design and involved the same population criteria and interventions, but the samples and outcome assessments differed. The core measure of service utilisation in Theme 1 was use of hospital services, to support a business care for the adoption of telemonitoring technologies in the United Kingdom. Due to the large skew in distribution of hospital use, a large sample of patients were required (see sample size calculation below). Therefore, all patients who receive telecare or telehealth in the practices involved in the evaluation were to have routine data on health and social care service extracted from existing data sources. This did not require the individual patient assessments required for Themes 2 and 3. Themes 2 and 3 used a subsample of patients who were assessed using patient reported outcome measures (PROMs) and health utilisation measures to provide a more comprehensive assessment of clinical and cost-effectiveness. The design is shown in more detail in Figure 6.

Data collection for Theme 1 will be based on extraction from existing operational information systems in a variety of health and social care settings. This requires not only the extraction of large population-based data sets, but also a method to link data using a pseudoanonymised identifier which protects an individual's identify in accord with national guidance.

### Population

#### (a) *Patients with long-term health conditions*

Three clinical conditions were included in the study: heart failure; diabetes and COPD. In the interests of maximising external validity in this pragmatic trial, eligibility was not conferred on the basis of formal clinical assessment of disease severity (e.g. HbA1c, FEV1 % predicted, brain natriuretic peptide test). Instead patients were deemed eligible on the basis of either (i) their inclusion on the relevant Quality Outcomes Framework (QOF) register in primary care, (ii) a confirmed medical diagnosis in primary or secondary care medical records as indicated by GP Read Codes or ICD-10 codes, or (iii) confirmation of disease status by a local clinician (i.e. GP, community matron) or by their hospital consultant. Patients were not excluded on the basis of additional physical co-morbidities.

#### (b) Individuals with social care needs

Inclusion criteria were informed by the Department of Health Fair Access to Care criteria, and included people aged 18+ meeting one or more of the following criteria: currently in receipt of, or considered to have a need for night sitting; receiving 10 or more hours per week of home care; receiving 1 or more days per week of day care; mobility difficulties; those who have had a fall or who are considered at high risk of falling; a live-in or nearby carer facing difficulties carrying their current burden of responsibilities; or cognitive impairment/confusion with live-in or nearby carer.

### Interventions and comparisons

The evaluation is designed as an assessment of the value of two types of telemonitoring technology broadly characterised as telehealth and telecare. Each site has separate agreements with technology suppliers and there is no attempt to standardise the exact technology across sites. The analysis plan does not propose to evaluate the effects of individual technologies and the study is not powered to do so.

#### (a) Telehealth and telecare

Telehealth participants in Cornwall and Kent received a home monitoring system comprising a base unit (the *Tunstall RTX 3370 *or the *Viterion V100 *respectively), which is a small device with an LCD screen and response buttons to allow navigation of symptom questions and educational messages to be transmitted to participants, together with up to four peripheral monitoring devices. In Newham, telehealth participants received the Philips Motiva Personal Healthcare System comprising a set top box that connects to a television allowing symptom questions, educational videos and a graphical history of recent clinical readings to be accessed via a dedicated channel, plus an equivalent range of peripheral monitoring devices. Sites used different protocols for allocating peripheral devices but across all sites the critical devices by condition were a pulse oximeter (for COPD), a glucometer (for diabetes) and weighing scales (for heart failure). Participants with multiple conditions received multiple peripheral devices. Participants were asked to take clinical readings up to 5 days per week at the same time each day but the frequency was adjusted according to their individual history (e.g. a participant with diabetes and well controlled blood glucose would be asked to take readings less frequently than one with poorly controlled blood glucose). In Cornwall and Newham the base unit or set top box provided a visual and audio reminder when readings were due. At the end of each session data from clinical readings and symptom questions were sent to a monitoring centre via a secure server either automatically (in Cornwall and Newham) or following participant authorisation (in Kent).

Across all sites, telecare participants received a *Tunstall Lifeline Connect *or *Connect+ *base unit and pendant alarm together with any number of 27 peripheral devices classified into four broad categories: personal health and well-being sensors (e.g. bed/chair occupancy sensors, enuresis sensor, epilepsy sensor, fall detector, medication dispenser), sensory impairment aids (e.g. big button telephone, wearable vibrating alert), safety and security aids (e.g. bogus caller button, key safe) and environmental monitoring sensors (e.g. carbon monoxide detector, heat sensor, flood detector). Peripheral telecare devices were allocated on the basis of a needs assessment and participants with multiple needs were allocated appropriate combinations of peripheral devices. Telecare is primarily a passive system to monitor behaviour (via sensors around the home), facilitate independent living (via aids for memory, safety and sensory impairment) and raise alarms either automatically (via sensors) or manually (via a personal alarm or pull cord) in the event of an emergency. As such there was no required behavioural regimen that telecare intervention participants were expected to adhere to (i.e. they were not expected to take any clinical readings or answer symptom questions on a regular basis via the base unit or set top box). Data from sensors and alarms were automatically sent to a monitoring centre via a telephone line.

Monitoring centres were staffed by specialist nurses and community matrons. Incoming telehealth readings were automatically classified using a traffic light system of red, amber or green alerts based on relevant NICE guidelines or on individually tailored criteria specified by clinicians familiar with the case history. Red alerts in telehealth usually represent an opportunity for early intervention to prevent or minimise imminent clinical deterioration (e.g. titration of medications in response to a sudden weight change in heart failure may prevent critical exacerbation of symptoms and avoid the need for more costly and/or more risky treatments). Incoming data from certain telecare sensors (e.g. fall detector, heat sensor, smoke alarm) generated a red flag if outside set parameters to indicate a potential emergency situation requiring an immediate response. Other telecare sensors (e.g. bed/chair occupancy sensors) generated more ambiguous information and usually required contact with the user to establish their status. Telehealth alerts were typically monitored during office hours on a daily basis (Monday to Friday), while telecare alerts were monitored 24 hours per day, 365 days per year. Monitoring centre staff provided a stepped-care response to alerts ranging from reviewing recent clinical readings but taking no further action or requesting a repeat reading (these responses are for telehealth only) through to contacting the participant via the base unit or telephone, visiting the participant or referring on to another healthcare professional (e.g. GP, secondary care services or emergency services).

#### (b) Waiting list controls

Telehealth and telecare participants randomised to the control arm received standard health and social care for 12-months and then, subject to re-assessment, were offered telehealth or telecare at the end of trial.

### Outcomes

Theme 1 will collect and link large, administrative datasets with the aim of tracking service use for (a) up to three years pre-intervention and (b) 12 months post- intervention. It will include an assessment of hospital utilisation, including emergency and elective inpatient admissions, inpatient bed day use, outpatient attendances and accident and emergency visits; primary care utilisation, including GP encounters, prescription drugs and community matron visits; and, if possible, social care utilisation such as the use of domiciliary and residential care. Costs will be attributed to activity using national Payment by Results tariffs and reference costs.

Theme 2 and 3 include a comprehensive assessment of PROMs and health utilisation measures at 3 and 12 months, including general quality of life [14-16], disease-specific quality of life (teleheath only) [17-19], psychological well being [20,21], perceived acceptability of telemonitoring devices; use of telehealth and telecare; attitudes, self-efficacy and self-care behaviours [22-25], clinical outcomes (for telehealth participants), social networks [26], illness burden [27], disability [28,29] and health care and social services utilisation [30,31]. Carer outcomes include carer anxiety and caregiver strain [32,33], and carer costs include carer time spent in providing care to the participant as well as lost productivity. The full range of assessment instruments is provided online (Additional file 1).

### Sample size calculation

#### (a) Sample size calculation for Theme 1

The primary outcome measure was taken as the proportion admitted to hospital, with the secondary outcome measure being number of bed days. It was thought important to be able to detect a relative change in admission proportion of between 15% and 20%, from a baseline of 25% (estimated from actual site data), and a change in bed day use of 20%, both at power of 80% and two-sided p-value of < 0.05. Previous studies in the older population suggested that the intra cluster correlation (ICC) for admissions would be around 0.001 [34]. Sample size calculations were carried out using appropriate formulae [35] and suggested that a sample of 3,000 patients would allow the detection of a relative risk reduction (RRR) of 17.5% in admission proportion and a 20% reduction in bed days using the above criteria. Given that two separate RCTs of telehealth and telecare were being run, this means that the overall target sample size for Theme 1 was 6,000 patients.

#### (b) Sample size calculation for Theme 2 and 3

The sample size requirements were calculated in relation to COPD using the most plausible condition-specific health related quality of life measure: the Chronic Respiratory Questionnaire (CRQ) and its key dimension (the dyspnoea scale). Baseline mean scores were estimated at between 2.3 and 3.3 with a standard deviation of 1.0 [36]. Taking the minimal clinical important difference (MCID) at a conservative 0.3 [37], the effect size (given the observed baseline SD of 1.0) would be 0.3. Since ICC values of around 0.03 for both morbidity [38] and health related quality of life [39] variables have been reported, the ICC was estimated at 0.01 and 0.05. With power of 80% and two-sided p-value of < 0.05, the required sample size would be between 200 and 300 per condition (i.e. an overall sample size of 900), assuming the effect size for the health related quality of life measures is around the same level of 0.3 for the other conditions.

### Trial procedures

General practices were approached by letter inviting them to take part in the trial. Once a practice had consented, potential participants for telehealth were identified in each site using existing registers of patients with long-term conditions in general practice. Potential participants for telecare in each practice were identified from databases held by social services departments.

To meet ethical obligations, patients were asked to complete and return a 'data sharing letter' if they consented to their data being shared with the research team. Once this letter was received, patients received a 'light touch' visit from a member of the project team in each site, where consent was taken to (a) participate in the main trial (Theme 1) and (b) the questionnaire study (Themes 2 and 3).

Participants who agreed to take part in the Themes 2 and 3 were subsequently contacted by a market research company to arrange a convenient time for the baseline interview. At this interview, patients received information about this part of the study and signed consent. The baseline assessment varied by participant status (i.e. COPD, heart failure, diabetes, telecare, carers) but each comprised a core of standardised PROMs. The PROMs were self-completed by the participant with the researcher on hand to explain or clarify. Data on health care utilisation were collected via interview. The average total time for assisted baseline interview completion was 80 minutes.

Carers of service users in the trial were identified by sites usually at the 'light touch' visit, either by the carer expressing an interest or via *snowball sampling *(i.e. asking participants if they had an informal carer at the baseline interview).

Each practice was allocated to groups via a centrally administered minimisation algorithm. The allocation determined the technologies available to each practice (i.e. either telehealth or telecare) and their associated patients. Following installation of the technologies, participants will be followed-up for 12-months. After 12-months, the 'usual care' groups were eligible to receive the appropriate interventions.

### Analysis

The overall trial essentially involves two nested trials (one comparing telehealth with usual care, and one comparing telecare with usual care) which will be analysed separately but using identical procedures.

Unlike other evaluations of technologies in the NHS [40], the trial is not primarily designed to assess the effectiveness of any particular type of telecare or telehealth technology, and the primary analysis is at the level of the technology type (i.e. telehealth or telecare). There are no plans to conduct analyses of individual technologies.

#### (a) Themes 1 and 3

Baseline characteristics (e.g. age; sex; ethnicity; co-morbidities; risk of hospitalisation; typical hospital use) of the patients in the intervention and usual care arms will be compared and adjusted for where appropriate. Utilisation (inpatient admissions, bed day use, and cost) will be compared between the two groups for the three years before each patient receives the intervention, and the 12 months afterwards using appropriate statistical models for cluster randomised trials.

One method of analysing the results of Theme 1 will be using a risk stratification tool, to identify individuals at different levels of risk of future hospitalisation. Two such tools will be used, the Patients At Risk of Rehospitalisation (PARR) model and the Combined Predictive Model.^2 ^Both of these models are in use in the NHS to case-find patients for admission-avoidance programmes. The aim will be to understand which patients saw greatest changes in service use from the addition of telehealth or telecare, according to standard metrics used routinely to target services in parts of the NHS.

Data on health, social care and other support service use for participants will be collected using the CSRI [30]. Unit costs will be attached to service use data to calculate a total cost per patient at baseline, 3 months and 12 month follow up. Costs will be calculated on the basis of the costs incurred in the 3 months prior to the 3 data collection points. Service use and associated costs for participants in both trial arms will be reported under sub-categories such as use of acute hospital services and community social services. All cost categories will be allocated to a perspective (whether NHS, Local authority, NHS and Local authority, or public sector). The costs of the intervention will be calculated specifically for this study from data provided by the sites and those costs attached to participants receiving the intervention in the telehealth and telecare groups. Point estimates of the costs for the control and intervention groups in each trial arm will be derived from univariate and multivariate analyses of costs from all 4 perspectives.

The effectiveness of the telehealth and telecare programmes will be compared using a common unit of outcome measurement, the QALY, to be constructed from the EQ-5D health state classification [16] scored using established algorithms (York Tariff) [41]. QALY scores will be calculated for the baseline, 3 month and 12 month follow-up points. In addition, scores from a broader measure of quality of life intended for health and social care evaluations, the ICECAP [14], will be examined. ICECAP scores at each time point will be presented for all three time points. Incremental cost effectiveness ratios (ICER) will be calculated at the 3 month and 12 month follow up points for both trial arms. The ICER will be defined as the difference in mean costs incurred by the treatment groups in each arm over the previous 3 month period, divided by the difference in mean QALY gain between those treatment groups. A cost-effectiveness acceptability curve (CEAC) will be produced, based on the results of a model of net monetary benefit for clustered data. The CEAC allows the decision-maker to see what is the likelihood of telehealth and telecare being cost-effective at different monetary values of increments of improvement in the chosen outcome.

#### (b) Theme 2

Clinical effectiveness (quality of life, well-being, self-care, and carer burden) at 3 and 12 months will be compared between the groups, taking into account scores at baseline, and adjusting for potential confounders where appropriate, using statistical models suitable for cluster randomised trials.

The telehealth trial involves three separate clinical populations, and we will assess the differential impact of telehealth in these different clinical populations using a pre-specified subgroup analysis across conditions (including a group with co-morbidities).

## Discussion

**Figure 7:**
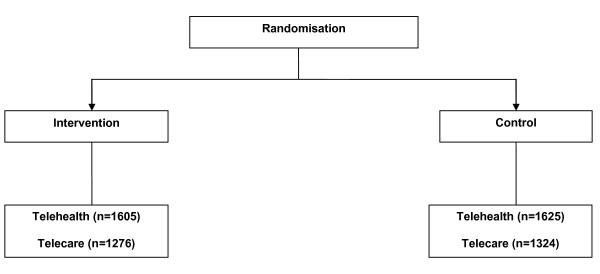
**CONSORT diagrams (baseline data only)**.

The proposed study is an ambitious project that is designed to provide a pragmatic yet rigorous assessment of the benefits of (a) telehealth in the management of patients with long-term health conditions, and (b) telecare in the management of patients with social care needs. Figure 7 shows the CONSORT diagram for the recruitment to the trial which shows that 97% of the patient sample size target has been achieved.

### Advantages and disadvantages of the proposed design

Adoption of the cluster design means that individual patients are not randomised to technology or usual care. This overcomes concerns raised by the participating sites, who felt that individual randomisation was unlikely to be acceptable to patients or professionals: in the adopted design, all general practices will have immediate access to the roll-out of telemonitoring equipment for at least some of their patients. The design thus overcomes concerns about resentful demoralisation among practices [42] if some practices had been randomised to usual care for all their patients, especially as all participants are assessed for the technology after 12 months. The current study is a cluster randomised design, using general practices as the unit of randomisation. However, the present design is an 'individual cluster' design [43]. This means that a cluster randomised design has been adopted for various reasons, but the intervention is still delivered to individual patients. This is unlike a 'cluster cluster' design, where patients in the cluster cannot refuse the intervention (e.g. a water fluoridation trial).

Clearly, the planned analysis by type of technology (telehealth and telecare) ignores differences within technologies. The study has not been powered to explore the effects of individual technologies, but combining different technologies into the primary analysis will lead to debates about their comparability [44]. The qualitative data from Theme 4 may be a useful source of data on variation among users and professionals in their experience of different technologies.

It is important to note that all practices taking part in the evaluation are part of the Whole System Demonstrator pilots, and thus all practices and patients are potentially benefiting from the wider service redesign that is ongoing in these sites. Therefore, the study is assessing the added value of telehealth and telecare technologies over and above the effects of this wider service redesign, and not the benefits of whole systems redesign versus conventional care (see Figure 1). There will therefore be issues concerning the generalisability of the results, because the sites which are part of the Whole System Demonstrator pilots have been specifically chosen for their innovations in these areas of care.

The themes address related issues using a range of methods. For example, Theme 1 exploits large, existing sources of administrative data on service use while Theme 3 collects self-reported data from patients. Several studies have compared health care utilization as described in administrative and self-reported data [45,46]. These have typically found significant differences, especially for people with high levels of use, older people, and for people with poor health status. The trial is also novel in the ways that it exploits data linkage across a range of operational data sets. The advantages of this approach are that it allows the team to access large volumes of computerised data relatively cheaply. This enables analyses of much larger groups but also includes historical data which can be used for risk adjustment and sub-group analyses. The disadvantages are that the data in administrative systems may be of poor quality and it may not capture the most important variables. The combination of administrative data (Theme 1), and directly recorded patient events (Theme 3) provide an opportunity to test whether data problems create different results.

The potential of telehealth and telecare technology to improve care and reduce costs is limited by a lack of rigorous evidence of actual impact. If telehealth and telecare technology proves cost-effective in our trial, it will provide additional options for health services worldwide to deliver care for populations with high levels of need.

## Competing interests

The authors declare that they have no competing interests.

## Authors' contributions

PB and MC drafted the manuscript and all other authors contributed to editing of the final manuscript. All authors read and approved the final manuscript

## Endnotes

^1 ^Originally a different design was planned. The funder wished to know the effects of the telehealth and telecare interventions, used alone and in combination for patients with health conditions *and *social care needs. The initial design was a complex factorial to allow estimation of the effects of (a) the individual technologies on patients with (i) long-term conditions (ii) social care needs and (b) the effects of the combination of technologies on patients with both long-term conditions and social care needs (see Additional file 2). In practice it was found that the majority of patients were exposed to one technology alone, and therefore the simpler design was eventually adopted.

^2 ^http://www.kingsfund.org.uk/current_projects/predicting_and_reducing_readmission_to_hospital/

## Pre-publication history

The pre-publication history for this paper can be accessed here:

http://www.biomedcentral.com/1472-6963/11/184/prepub

## Supplementary Material

Additional file 1 **Assessment instruments**. List of assessment instruments used in Theme 2 and 3. Click here for file

Additional file 2 **Original trial design**. A figure showing the original design of the trial. Click here for file
