# Supplementary material for: A comprehensive evaluation of the impact of telemonitoring in patients with long-term conditions and social care needs: protocol for the whole systems demonstrator cluster randomised trial
Source: BMC Health Serv Res. 2011 Aug 5;11:184. doi: 10.1186/1472-6963-11-184 (PMC3169462; doi:10.1186/1472-6963-11-184)
Supplement: Additional file 1 — Assessment instruments. List of assessment instruments used in Theme 2 and 3. [file 1472-6963-11-184-S1.DOC]

| **Scale** | **Items** | **Description** |
| --- | --- | --- |
| EQ-5D ^1^ | 6 | Generic preference-based measure of health for use in economic evaluations of health care |
| ICECAP ^2^ | 5 | Measure of quality of life for use in economic evaluation of health and social care interventions |
| ASCOT ^3^ | 2 | Adult Social Care Outcomes Toolkit measure of social care related quality of life |
| Client Services Receipt Inventory (CSRI) user and carer schedule ^4^ | 23  24 | Measure of service use, patterns of support from families, living arrangements, receipt of benefits and employment status (where relevant). |
| Brief STAI ^5^ | 6 | Measure of anxiety |
| CES-D 10 ^6^ | 10 | Measure of depression |
| UK SF12 ^7^ | 12 | Measure of health-related quality of life |
| HEIQ (selected sub-scales) ^8^ | 27 | Measures of quality of health education programs |
| MLHFQ ^9^ | 20 | Minnesota Living with Heart Failure Questionnaire measure of the effects of congestive heart failure on their lives |
| CRQ - Chronic Respiratory Questionnaire (adapted for study) ^10^ | 20 | Chronic Respiratory Questionnaire measure of quality of life for patients with chronic lung disease |
| DHP- Diabetes Health Profile (with additional subscale developed for the study) ^11^ | 18 | Measure of diabetes-specific quality of life |
| Generic Self-care Behaviours (developed for the study) | 6 | Measure of use of 6 recommended health scare behaviours |
| Self-care Behaviours Self Efficacy Scale (developed for the study) | 6 | Measure of confidence in performing self-care behaviours |
| EHFSCB – European Heart Failure Self Care Behaviours scale ^12^ | 12 | Measure of behaviour that heart failure patients perform to maintain life, healthy functioning, and well-being |
| COPD-SCB (developed for the study) | 24 | Measure of COPD self-care behaviours |
| SDSCA - Summary of Diabetes Self-Care Activities Measure (adapted for study) ^13^ | 11 | Measure of diabetes self-management |
| Generalized Self Efficacy Scale ^14^ | 10 | Measure of pptimistic self-beliefs to cope with a variety of difficult demands in life |
| Social Network Assessment Instrument ^15^ | 10 | Measure for identification of types of social network |
| Acceptability of telehealth and telecare (developed for the study) | 22 | Measure of participants’ beliefs and perceptions of telemonitoring equipment |
| Carer’s confidence and anxiety (developed for the study) | 13 | Measure of carer’s confidence and anxiety |
| Care-giver Strain Index ^16^ ^17^ | 13 | Measure of strain related to care provision |
| Illness Strain Index (developed for the study) | 9 | Measure of strain related to having a chronic illness - revision of Care-giver Strain Index for cared for person |
| Impact of Illness Scale ^18^ | 9 | Measure of the degree that illness/problems interferes with key roles and responsibilities in daily living |
| Townsend Disability Scale ^19^ ^20^ | 8 | Measure of activities that assesses physical ability in social terms |
| Subjective Norms ^21^ | 4 | Measure of social pressure to perform or not perform the target behaviour |

Reference List

1 Euroqol Copyright Group. Euroqol - a new facility for the measurement of health related quality of life. *Health Policy* 1990;**16**:199-208.

2 Coast J, Flynn T, Natarajan L, et al. Valuing the ICECAP capability index for older people. *Social Science & Medicine* 2008;**67**:874-882.

3 Netten A, Forder J, Shapiro J. *Measuring Personal Social Services Outputs for National Accounts: Services for older people (Discussion Paper No. 2267/3).* University of Kent, Canterbury. Personal Social Services Research Unit, 2006.

4 Beecham J, Knapp M. Costing psychiatric interventions. In: G Thornicroft, G Brewin, J Wing, eds. *Measuring Mental health Needs*. London: Gaskell, 2001;220-224.

5 Marteau T, Bekker H. The development of a six-item short-form of the state scale of the Spielberger State-Trait Anxiety Inventory (STAI). *British Journal of Clinical Psychology* 1992;**31**:301-306.

6 Andersen E, Malmgren J, Carter W, Patrick D. Screening for depression in well older adults: Evaluation of a short-form of the CES-D (Center for Epidemiologic Studies Depression Scale). *American Journal of Preventative Medicine* 1994;**10**:77-84.

7 Jenkinson C, Layte R, Jenkinson D, et al. A shorter form health survey: Can the SF-12 replicate results from the SF-36 in longitudinal studies? *Journal of Public Health Medicine* 1997;**19**:179-186.

8 Osborne R, Elsworth G, Whitfield K. The Health Education Impact Questionnaire (heiQ): An outcomes and evaluation measure for patient education and self-management interventions for people with chronic conditions. *Patient Education and Counseling* 2007;**66**:192-201.

9 Rector T, Kubo S, Cohn J. Patients self-assessment of their congestive heart failure: Content, reliability and validity of a new measure, the Minnesota Living with Heart Failure questionnaire. *Heart Failure* 1987;**3**:198-209.

10 Guyatt G, Berman L, Townsend M, Pugsley S, Chambers L. A measure of quality of life for clinical trials in chronic lung disease. *Thorax* 1987;**42**:773-778.

11 Meadows K, Abrams C, Sandbaek A. Adaptation of the Diabetes Health Profile (DHP-1) for use with patients with Type 2 diabetes mellitus: psychometric evaluation and cross-cultural comparison. *Diabetic Medicine* 2000;**17**:572-580.

12 Jaarsma T, Strömberg A, Mårtensson J, Dracup K. Development and testing of the European Heart Failure Self-Care Behaviour Scale. *The European Journal of Heart Failure* 2003;**5**:363-370.

13 Toobert D, Hampson S, Glasgow R. The summary of diabetes self-care activities measure: results from seven studies and revised scale. *Diabetes Care* 2000;**23**:943-950.

14 Schwarzer R, Jerusalem M. Generalized Self-Efficacy scale. In: J Weinman, S Wright, M Johnston, eds. *Measures in Health Psychology: A user's portfolio*. Windsor: NFER-Nelson, 1995;35-37.

15 Wenger G, Tucker I. Using network variation in practice: Identification of support network type. *Health and Social Care in the Community* 2011;**10**:28-35.

16 Robinson B. Validation of a caregiver strain index. *Journal of Gerontology* 1983;**38**:344-348.

17 Thornton M, Travis S. Analysis of the reliability of the Modified Caregiver Strain Index. *Journal of Gerontology* 2011;**58B**:S127-S132

18 Klimidis S, Minas H, Yamamoto K. Impact of Illness Scale: Reliability, validity and cross-cultural utility. *Comprehensive Psychiatry* 2001;**42**:416-423.

19 Townsend P. *Poverty in the United Kingdom*. Harmondsworth: Pelican, 1979.

20 Bowling A, Farquhar M, Browne P. Life satisfaction and associations with social network and support variables in the three samples of elderly people. *International Journal of Geriatric Psychiatry* 1991;**6**:549-566.

21 Francis J, Eccles M, Johnston M et al. *Constructing questionnaires based on the theory of planned behavior: A manual for researchers.* Newcastle. Centre of Health Services Research, 2004.
