# Supplementary material for: A comprehensive evaluation of the impact of telemonitoring in patients with long-term conditions and social care needs: protocol for the whole systems demonstrator cluster randomised trial
Source: BMC Health Serv Res. 2011 Aug 5;11:184. doi: 10.1186/1472-6963-11-184 (PMC3169462; doi:10.1186/1472-6963-11-184)
Supplement: Additional file 2 — Original trial design. A figure showing the original design of the trial. [file 1472-6963-11-184-S2.DOC]

**Randomisation of general practices to one of four groups**

**Group 2**

Patients with social care needs receive usual care

Patients with COPD, diabetes and CHD receive telehealth

Patients with social care needs and COPD, diabetes and CHD receive telecare

Patients with social care needs receive telecare

Patients with COPD, diabetes and CHD receive usual care

Patients with social care needs and COPD, diabetes and CHD receive telehealth

Patients with social care needs receive telecare

Patients with COPD, diabetes and CHD receive telehealth

Patients with social care needs and COPD, diabetes and CHD receive

usual care

Patients with social care needs receive usual care

Patients with COPD, diabetes and CHD receive usual care

Patients with social care and needs COPD, diabetes and CHD receive

telecare + telehealth

**Group 3**

**Group 1**

**Group 4**
